# Identifying Two Novel Clusters in Calcium Oxalate Stones With Urinary Tract Infection Using 16S rDNA Sequencing

**DOI:** 10.3389/fcimb.2021.723781

**Published:** 2021-11-17

**Authors:** Chen Shen, Qianhui Zhu, Fan Dong, Wei Wang, Bo Fan, Kexin Li, Jun Chen, Songnian Hu, Zilong He, Xiancheng Li

**Affiliations:** ^1^ Departmant of Urology, The Second Affiliated Hospital of Dalian Medical University, Dalian, China; ^2^ State Key Laboratory of Microbial Resources, Institute of Microbiology, Chinese Academy of Sciences, Beijing, China; ^3^ University of Chinese Academy of Sciences, Beijing, China; ^4^ Division of Biomedical Statistics and Informatics, Mayo Clinic, Rochester, MN, United States; ^5^ Beijing Advanced Innovation Center for Big Data-Based Precision Medicine, Interdisciplinary Innovation Institute of Medicine and Engineering, Beihang University, Beijing, China; ^6^ School of Engineering Medicine, Beihang University, Beijing, China

**Keywords:** urinary tract infection, 16S rDNA sequencing, differentially abundant taxa, calcium oxalate stones, urinary stone, microbiota

## Abstract

Urinary stones and urinary tract infection (UTI) are the most common diseases in urology and they are characterized by high incidence and high recurrence rate in China. Previous studies have shown that urinary stones are closely associated with gut or urine microbiota. Calcium oxalate stones are the most common type of urinary stones. However, the profile of urinary tract microorganisms of calcium oxalate stones with UTI is not clear. In this research, we firstly found two novel clusters in patients with calcium oxalate stones (OA) that were associated with the WBC/HP (white blood cells per high-power field) level in urine. Two clusters in the OA group (OA1 and OA2) were distinguished by the key microbiota Firmicutes and Enterobacteriaceae. We found that Enterobacteriaceae enriched in OA1 cluster was positively correlated with several infection-related pathways and negatively correlated with a few antibiotics-related pathways. Meantime, some probiotics with higher abundance in OA2 cluster such as Bifidobacterium were positively correlated with antibiotics-related pathways, and some common pathogens with higher abundance in OA2 cluster such as Enterococcus were positively correlated with infection-related pathways. Therefore, we speculated that as a sub-type of OA disease, OA1 was caused by Enterobacteriaceae and the lack of probiotics compared with OA2 cluster. Moreover, we also sequenced urine samples of healthy individuals (CK), patients with UTI (I), patients with uric acid stones (UA), and patients with infection stones (IS). We identified the differentially abundant taxa among all groups. We hope the findings will be helpful for clinical treatment and diagnosis of urinary stones.

## Introduction

Urinary stones are common urology diseases. Most urinary stones are composed of calcium oxalate (Evan, 2010). Uric acid stones and infection stones each account for approximately 10% of all urinary stones, and rare stones such as cystine stones account for approximately 1% (Wu et al., 2014). Epidemiological survey results in China showed that the prevalence of urinary stones reached 6.4% and that, in some areas, it could exceed 10% (Zeng et al., 2017). The recurrence rate of urinary stones ranges 6%–17% within 1 year, 21%–53% in 3 to 5 years, and 60%–80% in a lifetime (Liu et al., 2018).

The exact pathogenesis of urinary stones is unclear. There are many hypotheses but none are definitive. The incidence of urinary stones is related to age, gender, race, dietary habits, genetics, and metabolism (Sayer, 2017; Taguchi et al., 2017; Jung et al., 2018; Kachroo et al., 2021). Urinary stones are also closely associated with microbial taxa (Dornbier et al., 2020). For example, *Oxalobacter Allison* is known to maintain oxalate homeostasis in the gut. Ticinesi et al. found that this species was negatively associated with calcium oxalate stone formation (Ticinesi et al., 2018). Liu et al. found that several short-chain fatty acids (SCFAs) producing bacteria and related metabolic pathways were significantly reduced in the gut microbiota of patients with urinary stones (Liu et al., 2020). However, urinary microbiome has a great impact on urinary stones than the gut microbiome (Zampini et al., 2019). Dornbier et al. analyzed the potential relationships between OA and urinary microbiome using metagenomics next-generation sequencing (mNGS) technology (Dornbier et al., 2020). Xie et al. confirmed that male patients with urinary stones had different urinary microbiome compositions and significantly lower microbial diversity than healthy subjects (Xie et al., 2020). Stones and infections always occur simultaneously. The relationships between urine microbiota and urinary stones existed not only in IS but also in other urinary stone diseases (de Cogain et al., 2014). Mouse model studies showed that *Escherichia coli* was associated with crystal aggregation during calcium oxalate-induced renal lesions (Barr-Beare et al., 2015). Proteins isolated from specific *E. coli* strains may induce calcium oxalate crystal aggregation (Amimanan et al., 2017). Microbiota may also adhere to stone crystals by upregulating inflammatory proteins in the urine (Suen et al., 2010).

Urinary microbiome may be naively related to urinary stones and UTI, which are the two most common diseases in urology. 16S rDNA sequencing is a regular strategy for clinical microbiome research. We sequenced and analyzed urine samples from patients in the OA group and a small number of samples in other groups (CK, I, UA, and IS). The purposes of the present study were to construct the urinary microbiome profiling of OA and other groups and to identify the relationships between the differentially abundant taxa and metabolic pathways.

## Materials and Methods

### Study Design and Population

Patients with calcium oxalate stones (OA), as well as patients in other groups including healthy individuals (CK), patients with UTI (I), patients with uric acid stones (UA), and patients with infection stones (IS) were selected for this project from June 2019 to June 2020. The average age of all the patients was 53.61 ± 12.46. All the patients suffer from UTI except for CK group. The stone composition was analyzed by infrared spectroscopy. All individuals did not use antibiotics during the first month before admission. Clean mid-stream urine was collected by catheter. The main medical histories included diabetes, hypertension, and other metabolic diseases. Patients were enrolled based on the diagnostic criteria of clinical UTI (white blood cells > 5 per high-power field) and were classified into OA, IS, and UA according to the main components of stones (one component > 50%).

### Urine Sampling

Clean mid-stream urine samples were collected by the clean catch method into a sterile container using proper clinical collection procedures. Some of the urine samples were subjected to expanded quantitative urine culture (EQUC). The remaining samples were kept at −80°C and transported to the laboratory for DNA isolation. This study was conducted in accordance with the guidelines of the Helsinki Declaration and Rules of Good Clinical Practice. This study was approved by Ethics Committee of the Second Affiliated Hospital of Dalian Medical University in accordance with the ethical review of biomedical research involving human beings (dayilunkuaishen2020045), and the samples were taken from informed and consenting individuals.

### DNA Isolation

Genomic DNA was extracted from urine using validated protocols. Briefly, 1 ml of urine was centrifuged at 13,500 rpm for 10 min, and the resulting pellet was resuspended in 200 μl of filter-sterilized buffer consisting of 20 mM Tris-Cl (pH 8), 2 mM EDTA, 1.2% Triton X-100, and 20 g/ml lysozyme and supplemented with 30 l of filter-sterilized mutanolysin (5,000 U/ml; Sigma-Aldrich, St. Louis, MO, USA). The mixture was incubated for 1 h at 37°C, and the lysates were processed through the DNeasy blood & tissue kit (Qiagen, Valencia, CA, USA) according to the manufacturer’s protocol. The DNA was eluted into 50 μl of AE buffer (pH 8.0) and stored at 20°C.

### 16S rDNA Gene Library Generation and Sequencing

Sequencing was performed using a NovaSeq desktop sequencer (Illumina, San Diego, CA, USA). Variable region 4 (V4) of 16S rDNA gene in each DNA sample was amplified. Briefly, the 16S rDNA V4 region was amplified in a two-step nested PCR protocol using the universal 515F and 806R primers, which were modified to contain the Illumina adapter sequences. Amplicons were analyzed by gel electrophoresis and purified using the QIAquick gel extraction kit (Qiagen). Extraction- and PCR-negative controls were included in all steps to assess potential DNA contamination. DNA samples were diluted to 10 nM, pooled, and sequenced using the paired-end 2 × 250 bp mode. Given that the urinary tract is a low biomass system and that 16S rDNA gene sequencing is highly sensitive, any contamination of the working space or sample may lead to skewed results. In an effort to limit false positives, therefore, controls were routinely utilized during processing.

### Bioinformatics Analysis

Barcodes and primers were removed from the 16S rDNA sequencing data using in-house scripts. The total effective reads were processed with QIIME2 (Hall and Beiko, 2018). Quality control and de-multiplexing of sequence data were performed with DADA2 plugins. Alpha and beta diversity analyses were computed by q2-diversity plugin on QIIME2. The Principal Coordinates Analysis (PCoA) was performed by vegan package in R software. Taxonomic assignment was performed using the classify-sklearn Bayesian methodology by q2-feature-classifier plug-in with the Greengenes database (DeSantis et al., 2006). Due to the low biomass nature of urine, the threshold for sequence positivity was set at a conservative cutoff of 10,242 sequence reads, and all samples were used in further analysis. The LEfSe was applied to determine the microbial taxa with significantly differential abundance between groups (LDA > 3.0) (Segata et al., 2011). Metabolic pathways were predicted by PICRUSt2 using the KEGG database (Douglas et al., 2020). The co-abundance networks of microbial taxa were visualized by Cytoscape version 3.72 (Shannon et al., 2003).

### Statistical Analyses

Statistical analyses were performed using R scripts. The Wilcoxon rank-sum test and Fisher’s exact test were used to compare clinical indexes among groups. The Wilcoxon rank-sum test was also used to compare the abundance of metabolic pathways. Spearman’s rank correlation test was used to calculate the correlation coefficients between the abundance of microbial taxa and metabolic pathways. The interactions of co-abundance microbial taxa were calculated by Spearman’s rank correlation test.

## Results

### Clinical Characteristics of All Cohorts

In total, 107 samples of five groups (OA = 69, I = 8, IS = 16, UA = 9, and CK = 5) were collected (**Table S1**). The average age of total samples was 53.61 ± 12.46, including 65 men and 42 women. There were no significant differences in age, maximum body temperature in the perioperative period, WBC (white blood cell) in blood, and pH of urine among the five groups (*p* > 0.05). The routine urine examination showed that the bacterial concentration and WBC/HP (white blood cells per high-power field) in the CK group were significantly lower than those in other groups (*p* < 0.05).

### 16S rDNA Sequencing and Bioinformatics Analysis

**Figure 1:**
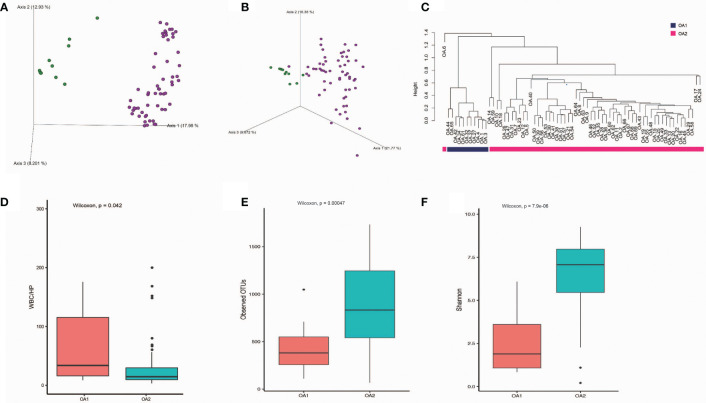
The differences of OA1 and OA2 clusters in urinary microbiota composition and the urine WBC/HP index. **(A)** Beta diversity analysis of OA1 and OA2 clusters in Bray-Curtis distance method. Green dots represent OA1 samples, purple dots represent OA2 samples. **(B)** Beta diversity analysis of OA1 and OA2 clusters in Jaccard distance method. Green dots represent OA1 samples, purple dots represent OA2 samples. **(C)** Hierarchical clustering based on OTU relative abundance of OA1 and OA2 samples. **(D)** The distribution of urine WBC/HP index between OA1 and OA2 clusters. **(E)** The distribution of observed OTUs between OA1 and OA2 clusters. **(F)** The distribution of Shannon index between OA1 and OA2 clusters.

We obtained 57,768 ± 17,567 high-quality reads from each sample after quality control. Upon further analysis, according to criteria stipulated in the methods, we obtained 43,080 OTUs in all samples, from 283 species, 714 genera, 385 families, 198 classes, 320 orders, 75 phyla, and 2 kingdoms (Bacteria and Archaea). The rarefaction curves reached a plateau, indicating that the bacterial diversity present in every sample had been satisfactorily detected (**Figure S1**). According to **Figure S2**, the top three phyla with the highest abundance were Proteobacteria, Firmicutes, and Cyanobacteria, which account for 55.7%, 16.3%, and 6.6%, respectively (**Figure S2**). The beta diversity implied that the OA group was divided into two clusters, while the other groups were not clearly differentiated (**Figure S3**). In further analysis, beta diversity based on Bray–Curtis and Jaccard distance similarity matrix of the OA group verified this result (**Figures 1A, B**). Based on the relative abundance of microbial taxa in the OA group, we clustered the samples using a hierarchical clustering algorithm based on Euclidean distance and complete clustering method, and the result was consistent with the beta diversity plot (**Figure 1C**). Based on these results, we divided the OA group into two clusters (11 samples of OA1 and 58 samples of OA2) for further analysis.

### OA1 and OA2 Clusters Have Significant Correlations With WBC/HP

We initially focused on the clinical indexes of OA1 and OA2 clusters, and found that there were no significant differences between OA1 and OA2 clusters in age, gender, uric acid, and urine pH (**Table 1**). The urinary WBC/HP of OA1 cluster was significantly higher than that of the OA2 cluster (**Figure 1D**) (*p* < 0.05). The result suggested that the infection of OA1 samples was more serious than that of OA2 samples.

**Table 1:** The clinical indexes of OA1 and OA2 clusters.

Clinical indexes	OA1	OA2	Fisher *p*-value	Kruskal-Wallis *p*-value
Gender (Male/Female)	7/4	38/20	1.00	-
Age	54.27 ± 11.31	53.34 ± 11.49	-	0.69
Frist calculus (Yes/No/NA)	8/3/0	40/18/0	1.00	-
Repeated urinary tract infection (Yes/No/NA)	2/9/0	9/49/0	1.00	-
Preoperative antibiotics used (Yes/No/NA)	0/11/0	5/50/3	0.58	-
Upgrade antibiotics in peri operation period (Yes/No/NA)	2/9/0	1/55/2	0.07	-
The highest body temperature in peri-operation period	37.37 ± 0.86	37.29 ± 0.57	-	0.88
**Blood tests (NA)**				
WBC	7.57 ± 2.17	7.58 ± 4.43	-	0.39
PCT	0.05 ± 0.02	1.15 ± 7.44(10)	-	0.44
UA	383.69 ± 131.36	368.13 ± 110.13	-	0.65
K	3.95 ± 0.41	4.15 ± 0.47	-	0.20
Na	141.46 ± 1.44	140.92 ± 2.41	-	0.61
Ca	2.29 ± 0.10(1)	2.33 ± 0.13(5)	-	0.35
Mg	0.85 ± 0.08(1)	0.84 ± 0.08(5)	-	0.29
P	1.12 ± 0.15(1)	1.17 ± 0.18(5)	-	0.32
**Routine urine (NA)**				
Bacteria	1729.14 ± 4392.97	209.63 ± 265.80	-	0.27
WBC/HP	153.57 ± 298.90	81.72 ± 313.16	-	0.04
PH	6.23 ± 0.34	6.14 ± 0.57	-	0.73
**Urine culture**				
Drug resistant (Yes/No/NA)	2/9/0	3/52/3	0.19	-

### The OA1 Cluster Has Higher Abundance of Enterobacteriaceae Than the OA2 Cluster

**Figure 2:**
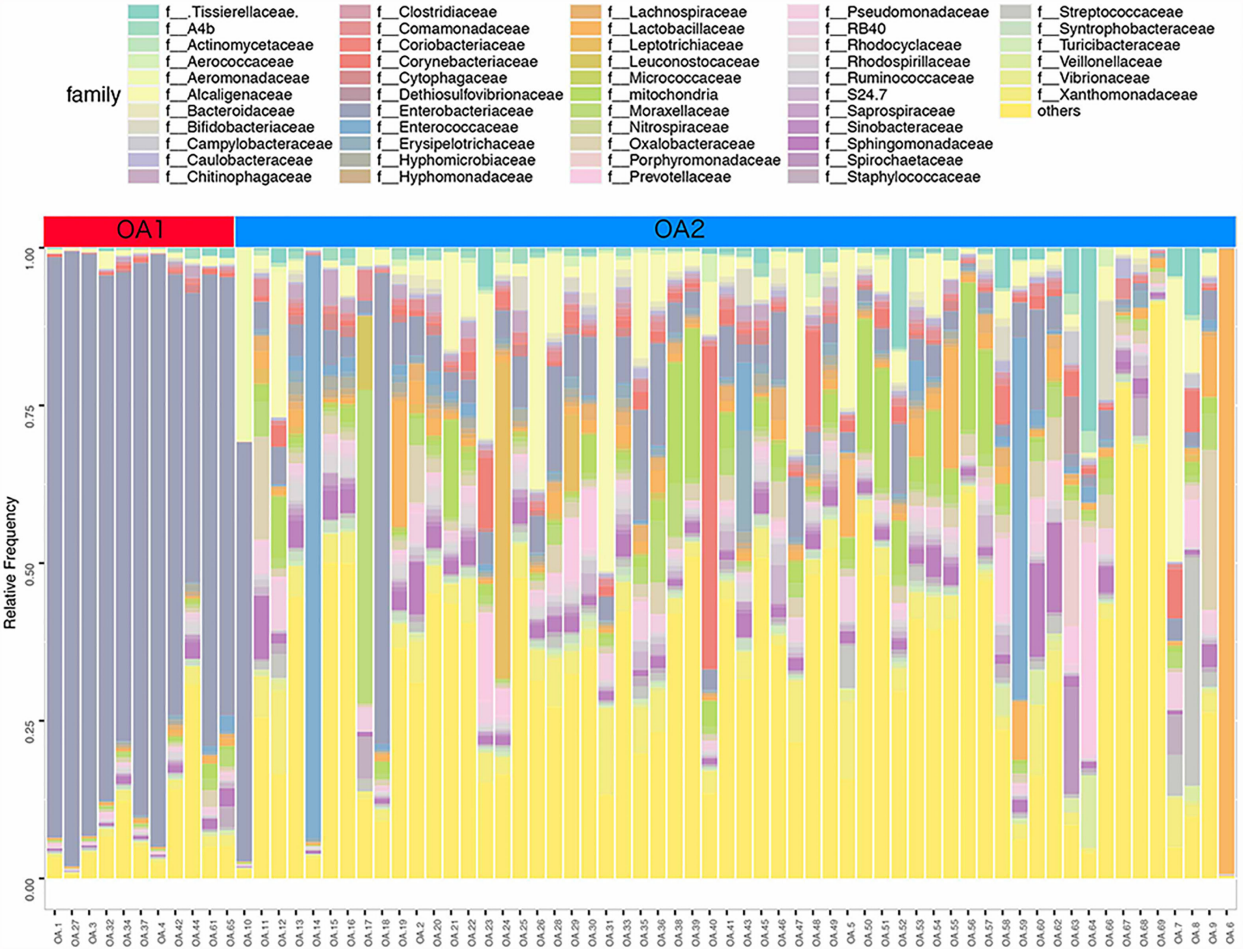
Stacked bar chart of the family level classification (top 50 of relative abundance) of urinary microbiota between OA1 and OA2 clusters.

In further analysis, we analyzed the microbiota compositions of OA1 and OA2 clusters. Alpha diversity results (**Figures 1E, F**) showed that the observed OTUs and Shannon diversity indexes of the OA2 cluster were significantly higher than those of the OA1 cluster (*p* < 0.05). Taxonomic classification results showed that, at the family level, the relative abundance of Enterobacteriaceae was the highest, accounting for 80.1% of each sample on average in the OA1 cluster, while the dominant microbial taxa of the OA2 cluster were not obvious. The microbiota diversity in the OA2 cluster was stronger than that in the OA1 cluster (**Figure 2**).

**Figure 3:**
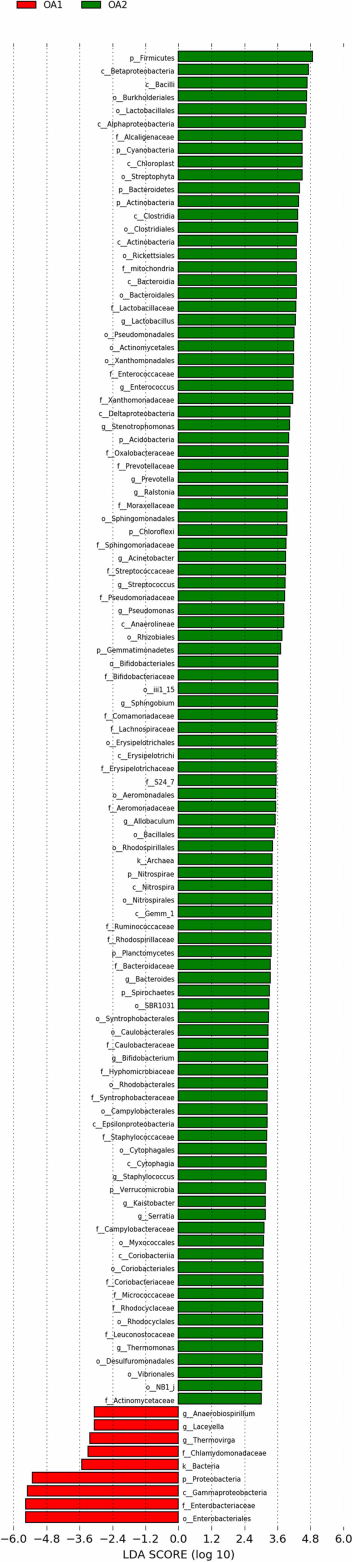
Differentially abundant taxa between OA1 and OA2 clusters in LEfSe analysis (LDA > 3.0).

There were 112 differentially abundant taxa between OA1 and OA2 clusters. Of these, nine microbial taxa had higher abundance in the OA1 cluster and 103 microbial taxa had higher abundance in the OA2 cluster (**Figure 3**). Compared to the OA2 cluster, the relative abundance of Proteobacteria and Enterobacteriaceae was significantly higher in the OA1 cluster. At the phylum level, the urinary tract microbiota of the OA2 cluster was mainly composed of Proteobacteria and Firmicutes. Unlike OA1, the OA2 cluster showed a higher diversity of Proteobacteria. The opportunistic pathogens in the OA2 cluster included *Staphylococcus*, *Acinetobacter*, Pseudomonadaceae, *Enterococcus*, and *Bacteroides* (Conti et al., 2009; Patrick et al., 2009; Howard et al., 2012; Jiang et al., 2012; Gao et al., 2018). The relative abundance of the probiotics *Bifidobacterium* and *Lactobacillus* was significantly increased in the OA2 cluster. *Prevotella*, which can break down plant dietary fibers in the human gut, was also significantly more abundant in the OA2 cluster.

### Enterobacteriaceae Enriched in the OA1 Cluster Was Positively Correlated With Multiple Infection-Related Pathways

**Figure 4:**
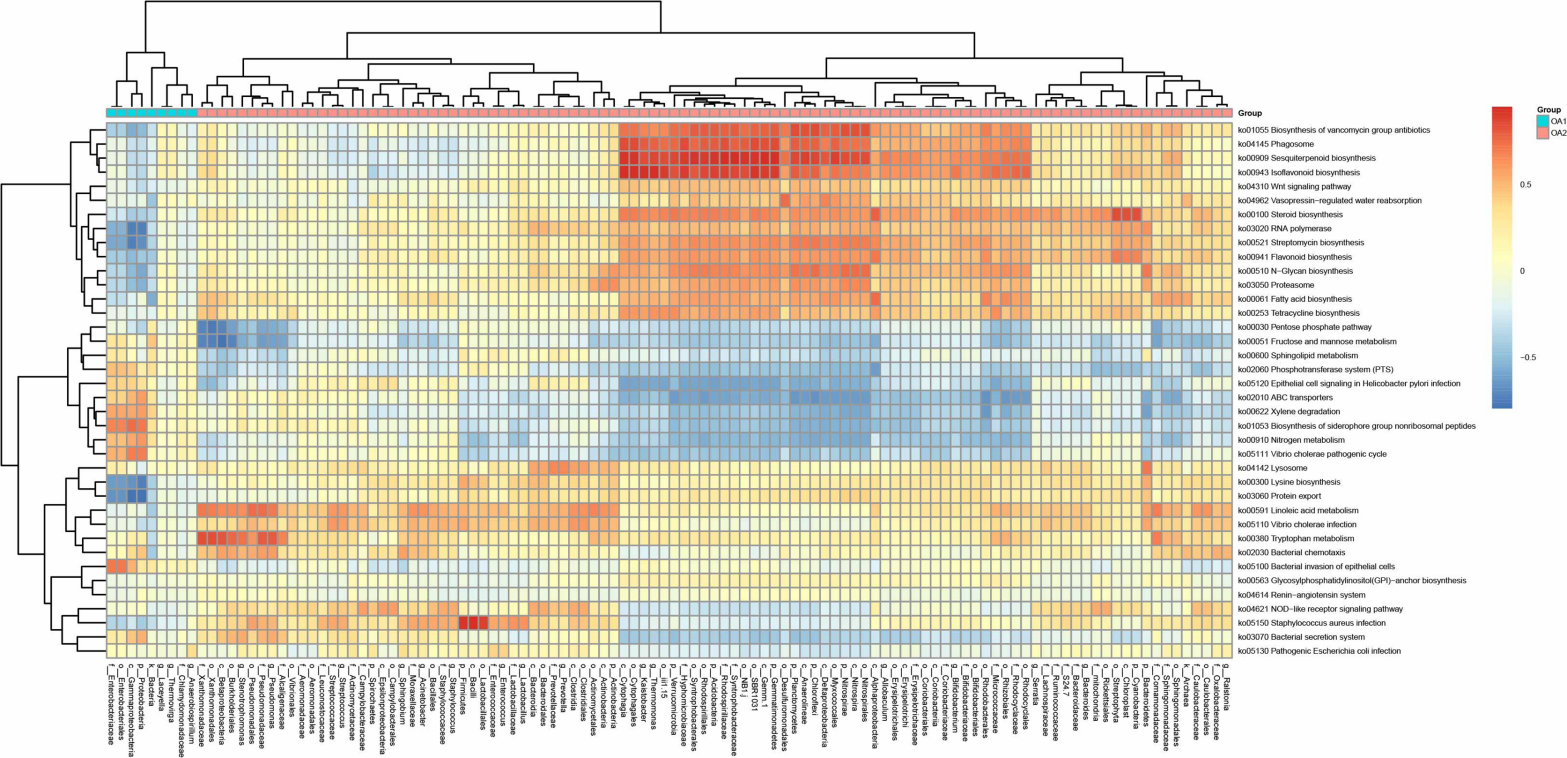
Correlation heat map between metabolic pathways and differential taxa between OA1 and OA2 clusters.

We performed PICRUSt analysis to predict the genetic potentials of the urine microbiota based on 16S rRNA sequences (**Figure 4**). Among all the differentially abundant taxa between OA1 and OA2 clusters, the highly abundant bacteria in the OA1 cluster, Enterobacteriaceae, had significantly positive correlations with infection-related pathways such as Xylene degradation (rho = 0.55, *p* < 0.05), Nitrogen metabolism (rho = 0.48, *p* < 0.05), *Vibrio cholerae* pathogenic cycle (rho = 0.53, *p* < 0.05), Biosynthesis of siderophore group nonribosomal peptides (rho = 0.67, *p* < 0.05), and Bacterial invasion of epithelial cells (rho = 0.71, *p* < 0.05). At the same time, Enterobacteriaceae was significantly negatively correlated with Lysine biosynthesis (rho = −0.64, *p* < 0.05), Protein export (rho = −0.67, *p* < 0.05), Streptomycin biosynthesis (rho = −0.58, *p* < 0.05), RNA polymerase (rho = 0.54, *p* < 0.05), and Biosynthesis of vancomycin group antibiotics (rho = 0.41, *p* < 0.05). Compared with the OA1 cluster, the highly abundant differential bacteria in the OA2 cluster, including *Enterococcus*, *Staphylococcus*, *Acinetobacter*, *Streptococcus*, *Pseudomonas*, and *Stenotrophomonas*, were significantly positively correlated with *Staphylococcus aureus* infection, *Vibrio cholerae* infection, and Linoleic acid metabolism (rho > 0.40, *p* < 0.05). Compared with OA1, other highly abundant different bacteria in the OA2 cluster, including *Bifidobacterium*, *Allobaculum*, *Thermomonas*, and *Kaistobacter*, were significantly positively correlated with Biosynthesis of vancomycin group antibiotics, Sesquiterpenoid biosynthesis, Isoflavonoid biosynthesis, and Steroid biosynthesis pathways (rho > 0.55, *p* < 0.05). In summary, we found that Enterobacteriaceae enriched in the OA1 cluster was positively correlated with multiple infection-related pathways, and there were no probiotics enriched in the OA1 cluster. The high-abundance bacteria in the OA2 cluster were divided into two parts. Among them, common conditional pathogens such as *Enterococcus* and *Staphylococcus* were significantly positively correlated with infection-related pathways. In addition, several probiotics such as *Bifidobacterium* were significantly positively correlated with antibiotic-related pathways (Padilha et al., 2019). In conclusion, the above reasons may have contributed to the higher infection of OA1 than OA2 cluster.

### The Differential Microbial Taxa in Co-Abundance Networks Display Different Patterns in OA1 and OA2 Clusters

**Figure 5:**
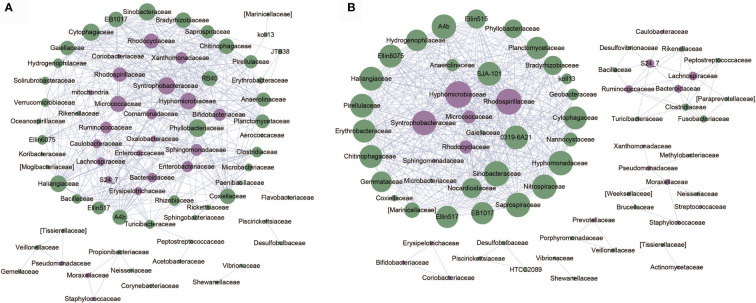
Co-abundance networks of microbial taxa of OA1 and OA2 clusters. The purple dots represent the differentially abundant taxa. The green dots represent the taxa whose abundance are not differential between OA1 and OA2 clusters. The size of the dots represents the degree. The links represent the interactions of microbial taxa. **(A)** Co-abundance networks of OA1; the correlations with |rho| > 0.8 are shown. **(B)** Co-abundance networks of OA2; the correlations with |rho| > 0.7 are shown.

Based on relative abundance of microbial taxa at the family level, we constructed the co-abundance networks of OA1 and OA2 clusters (**Figure 5**). The co-abundance network of OA1 cluster consisted of a larger sub-network centered on differentially abundant taxa and a smaller sub-network dominated by Pseudomonadaceae. Enterobacteriaceae was significantly more abundant (LDA > 3.0) in OA1 than in the OA2 cluster. In the larger sub-network of OA1 cluster, the bacterium showed highly significant negative correlation (rho < −0.8, *p* < 0.05) with several bacteria that were significantly more abundant (LDA > 3.0) in the OA2 cluster (**Figure 3**). These included Bifidobacteriaceae, Ruminococcaceae, and Micrococcaceae. The bacteria that were more enriched in the OA2 cluster were significantly positively correlated with each other, implying strong interactions between them (rho > 0.8, *p* < 0.05). There were possible inhibitory relationships between Enterobacteriaceae and Xanthomonadaceae and Sphingomonadaceae, while Xanthomonadaceae and Sphingomonadaceae had a mutually enhancing effect. Moraxellaceae, Pseudomonadaceae, and Staphylococcaceae were significantly positively correlated in the OA2 cluster network (rho > 0.8, *p* < 0.05), and these bacteria had significantly higher abundance in the OA2 cluster.

The co-abundance network of the OA2 cluster was composed of several smaller sub-networks and a larger sub-network dominated by differentially abundant taxa such as Hyphomicrobiaceae, Syntrophobacteraceae, and Rhodospirillaceae. The relative abundance of all differential bacteria in the OA2 cluster was significantly higher than those in the OA1 cluster (**Figure 3**). Among them, in the larger sub-network, Hyphomicrobiaceae was positively correlated with Rhodospirillaceae, Syntrophobacteraceae, Micrococcaceae, and Rhodocyclaceae (rho > 0.7, *p* < 0.05). In the smaller sub-network, Bifidobacteriaceae, Coriobacteriaceae, and Erysipelotrichaceae were significantly positively correlated with each other (rho > 0.7, *p* < 0.05). Moraxellaceae was positively correlated with Pseudomonadaceae and Staphylococcaceae (rho > 0.7, *p* < 0.05). Ruminococcaceae, Lachnospiraceae, and Bacteroidaceae showed significantly positive correlations (rho > 0.7, *p* < 0.05). In general, the hub nodes of OA1 and OA2 networks were similar, which implied that OA1 and OA2 were just two clusters of the OA group rather than two different diseases. However, in the OA1 network, Enterobacteriaceae was negatively correlated with most differential microbial taxa (rho < −0.7, *p* < 0.05). We therefore speculated that the different pathogenic bacteria lead to the difference of urine WBC/HP between OA1 and OA2 clusters. Specifically, the UTI of samples in the OA1 cluster was mainly caused by Enterobacteriaceae and the UTI of samples in the OA2 cluster was mainly caused by several pathogenic bacteria such as Pseudomonadaceae.

## Discussion

In this study, we focused on analyzing the urinary microbiota in the OA group and found that the high abundance bacteria in OA1 and OA2 clusters were different. Only Enterobacteriaceae predominated in the OA1 cluster, while the composition of highly abundant bacteria in the OA2 cluster was more diverse. Notably, there was a significant difference in urinary WBC/HP between OA1 and OA2 clusters, which may be due to the difference in the composition of the highly abundant microbial taxa. Previous studies have focused on the composition and abundance of urinary microbiota in different body parts (kidney stones, urinary stones, and bladder stones) and did not systematically distinguish the composition of urinary microbiota from different stone components (Dornbier et al., 2020). The present study is the first report to show that the OA group contains two clusters with differential urine WBC/HP index.

We analyzed the differentially abundant taxa between OA1 and OA2 clusters. The urine microbiota of OA2 samples had greater diversity, which included many common pathogens such as *Acinetobacter*, *Pseudomonas*, and *Enterococcus* belonging to different phyla and families. In contrast, the dominant microbial taxa of OA1 cluster was Enterobacteriaceae (over 80% on average), which contained several pathogens such as *E. coli*, *Klebsiella pneumoniae*, and *Salmonella enterica*. These bacteria are often reported to be related to UTI, intestinal inflammation, and multi-drug resistance and are the most common nosocomial pathogens (Naboka et al., 2021). In terms of clinical indexes, the urine WBC/HP level of the OA1 cluster was much higher than the OA2 cluster, which may be due to the differential relative abundance of Enterobacteriaceae in OA1 and OA2 clusters. From the perspective of metabolic pathways, Enterobacteriaceae was closely related to Bacterial invasion of epithelial cells (rho = 0.73) and Biosynthesis of siderophore group nonribosomal peptides (rho = 0.64), while these two pathways are considered related to UTI (Amalaradjou et al., 2011; Swayambhu et al., 2021). These pathways were negatively correlated with bacteria that had higher abundance in the OA2 cluster compared with the OA1 cluster (such as *Enterococcus*, *Staphylococcus*, and *Acinetobacter*). The results indicated that the dominant microbial taxa were closely related to UTI, and these pathways may be the mechanism leading to the high urinary WBC/HP in the OA1 cluster. Oxalobacteraceae, probiotics *Bifidobacterium*, and *Lactobacillus* were significantly different in OA1 and OA2 clusters (LDA > 3.0). In summary, we inferred that the OA1 cluster appears to be a sub-type of OA disease caused by the higher abundance of Enterobacteriaceae and the lack of probiotics.

We compared the differentially abundant taxa between OA1 and OA2 clusters and other groups (CK, I, IS, and UA) (**Figures S4**, **S5**). There were a total of 27 taxa shared between OA1 vs. CK and OA2 vs. CK. Among them, *Bacteroides*, Staphylococcaceae, Burkholderiaceae, and Enterobacteriaceae were considered to be taxa related to UTI. It was worth mentioning that, except for Enterobacteriaceae with higher abundance in OA1 and OA2 clusters, all other taxa had higher abundance in the CK group. Enterobacteriaceae are common pathogens of calcium oxalate stones and UTI (Tumturk et al., 2019). There were five shared taxa between OA1 vs. I and OA2 vs. I, mainly from *Fibrobacter*. There were 14 taxa in common between OA1 vs. IS and OA2 vs. IS. Consistent with the Venn diagram results of OA1, OA2, and CK groups, *Burkholderiales* was differentially abundant in OA1 vs. IS and OA2 vs. IS, and it can cause bacteremia, UTI, respiratory tract infection, and other infectious diseases. In addition, Enterobacteriaceae and *Proteus* were also differentially abundant in OA1 vs. IS and OA2 vs. IS, and these bacteria were highly abundant in the IS group. *Proteus* can produce urease, which can decompose urea into ammonia and carbon dioxide and produce highly alkaline urine, which is conducive to the crystallization of magnesium ammonium phosphate (Flannigan et al., 2018). There were 11 differential bacteria between OA1 vs. UA and OA2 vs. UA. *Ralstonia*, *Brevundimonas*, and *Proteus* were more typical clinical pathogens, and these bacteria were all highly abundant in the UA group.

This study demonstrated the capacity of 16S rDNA sequencing for clinical applications. Compared to traditional methods of isolation and culture, 16S rDNA sequencing is an *in situ* processing technology that can accurately reflect the microbiota composition of the sample with higher sensitivity. Compared to mNGS technology, less DNA volume is required for 16S rDNA sequencing, and it is not susceptible to host DNA contamination. However, because of the targeted sequencing technology, it is difficult for 16S rDNA sequencing to detect microbial taxa at species or strain level, and this is a challenge for the identification of pathogenic bacteria. In addition, 16S rDNA sequencing cannot accurately predict the gene functions of pathogenic bacteria, which hinders the study of disease etiology.

We found that the sample sizes of clusters OA1 and OA2 were not equal, which may be due to sampling bias or the lower prevalence of the OA1 cluster compared to the OA2 cluster. The results showed that OA1 was the more infectious cluster of the OA group, which may be caused by the higher abundance of Enterobacteriaceae. The urinary tract microbiome of the OA2 samples had a higher diversity of probiotics and pathogenic bacteria, and the degree of infection of OA2 samples was less than that of the OA1 samples. Our results may be helpful for early diagnosis and treatment of OA patients, and could aid the selection of drugs for clinical treatment.

## Data Availability Statement

The datasets presented in this study can be found in online repositories. The names of the repository is National Microbiology Data Center and the accession number is NMDC10017718.

## Ethics Statement

The studies involving human participants were reviewed and approved by the Ethics Committee of the Second Affiliated Hospital of Dalian Medical University (dayilunkuaishen2020045). The patients/participants provided their written informed consent to participate in this study. Written informed consent was obtained from the individual(s) for the publication of any potentially identifiable images or data included in this article.

## Author Contributions

XL and ZH accepted and designed research. CS, WW, and BF performed experiments. QZ, FD, KL, and SH analyzed the microbial sequencing data. CS, QZ, XL, and ZH wrote the manuscript. All authors contributed to the article and approved the submitted version.

## Conflict of Interest

The authors declare that the research was conducted in the absence of any commercial or financial relationships that could be construed as a potential conflict of interest.

## Publisher’s Note

All claims expressed in this article are solely those of the authors and do not necessarily represent those of their affiliated organizations, or those of the publisher, the editors and the reviewers. Any product that may be evaluated in this article, or claim that may be made by its manufacturer, is not guaranteed or endorsed by the publisher.

## Supplementary Material

Supplementary Table 1 Basic statistics of 107 urine samples.

Supplementary Figure 1 Rarefaction curves of observed OTUs in the 16S rDNA libraries.

Supplementary Figure 2 Histogram of top 50 relative abundance phyla found in each sample of 5 groups.

Supplementary Figure 3 Beta diversity plot based on abundance of OTUs in all samples of 5 groups. Green, purple, orange, blue and red represents OA, UA, IS, I and CK, respectively.

Supplementary Figure 4 Venn diagrams of differentially abundant taxa among OA1, OA2 and one of CK, I, IS, and UA group. **(A)** CK vs. OA1 and CK vs. OA2; **(B)** I vs. OA1 and I vs. OA2; **(C)** IS vs. OA1 and IS vs. OA2; **(D)** UA vs. OA1 and UA vs. OA2.

Supplementary Figure 5 Differentially abundant taxa for comparison of 4 groups (CA, I, IS, OA and UA) and OA1/OA2 in pairs. **(A)** CK and OA1; **(B)** IS and OA1; **(C)** I and OA1; **(D)** UA and OA1; **(E)** CK and OA2; **(F)** IS and OA2; **(G)** I and OA2; **(H)** UA and OA2.
