# Supplementary figures and images for: Identifying Two Novel Clusters in Calcium Oxalate Stones With Urinary Tract Infection Using 16S rDNA Sequencing
Source: Front Cell Infect Microbiol. 2021 Nov 17;11:723781. doi: 10.3389/fcimb.2021.723781 (PMC8635737; doi:10.3389/fcimb.2021.723781)

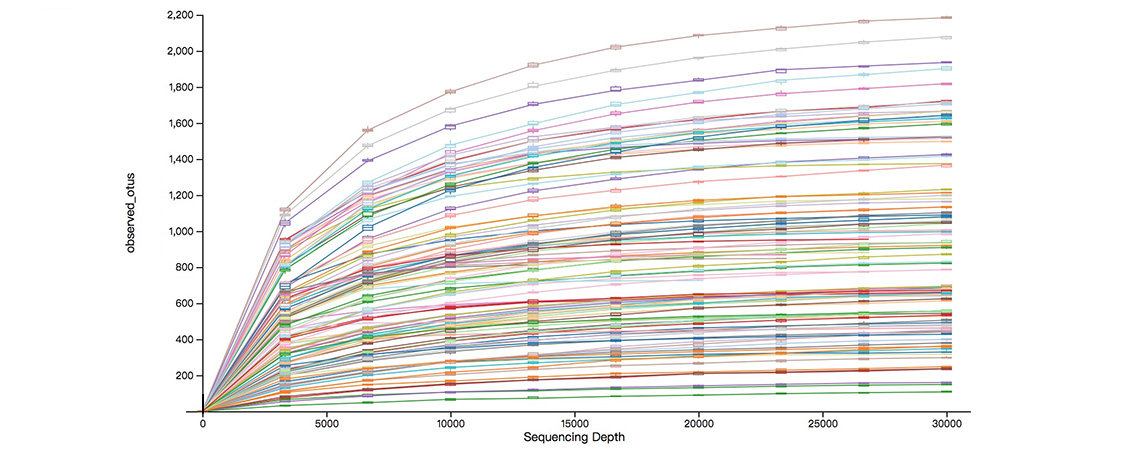

Supplement: Supplementary Figure 1 — Rarefaction curves of observed OTUs in the 16S rDNA libraries. [file Image_1.jpeg]

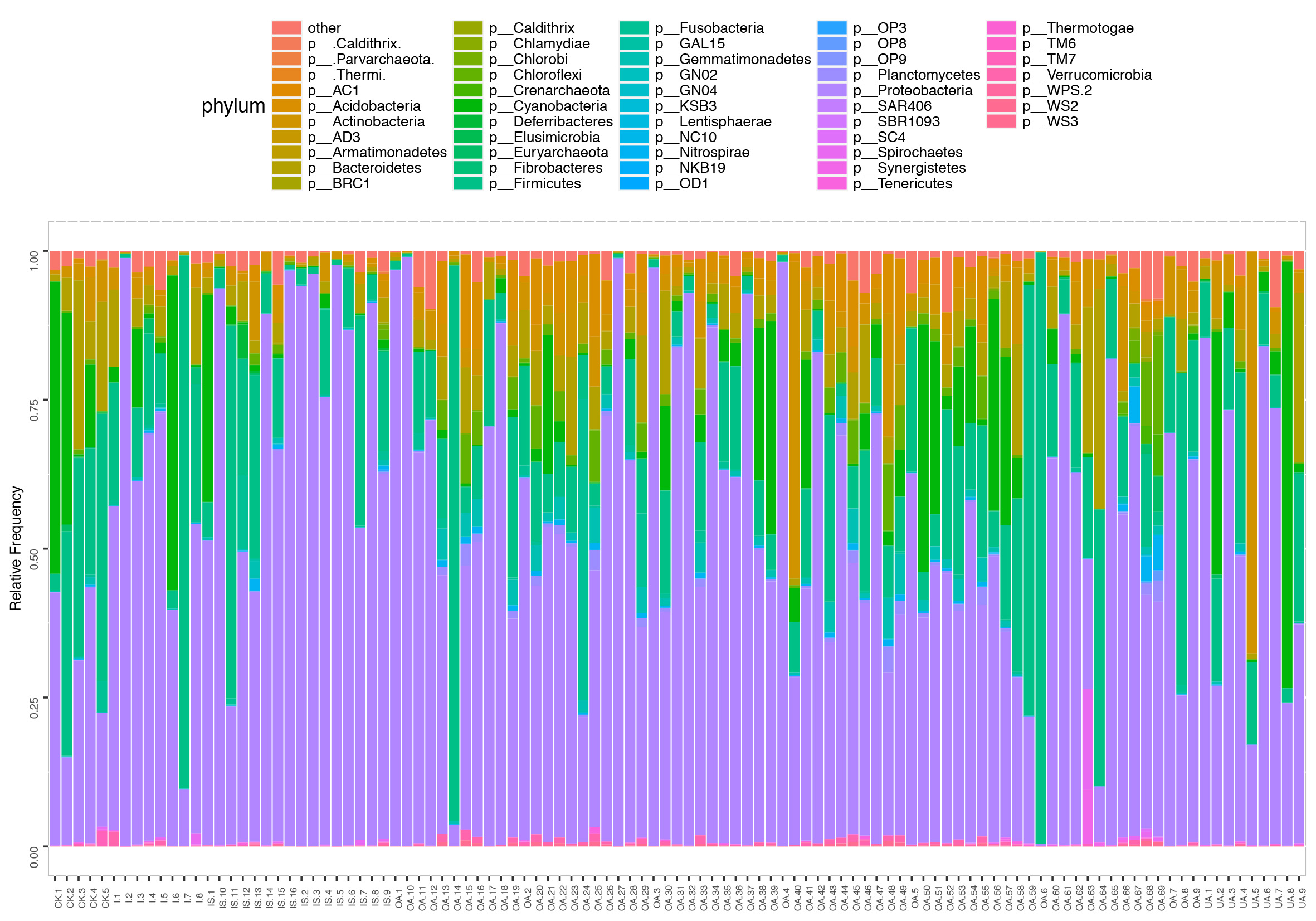

Supplement: Supplementary Figure 2 — Histogram of top 50 relative abundance phyla found in each sample of 5 groups. [file Image_2.jpeg]

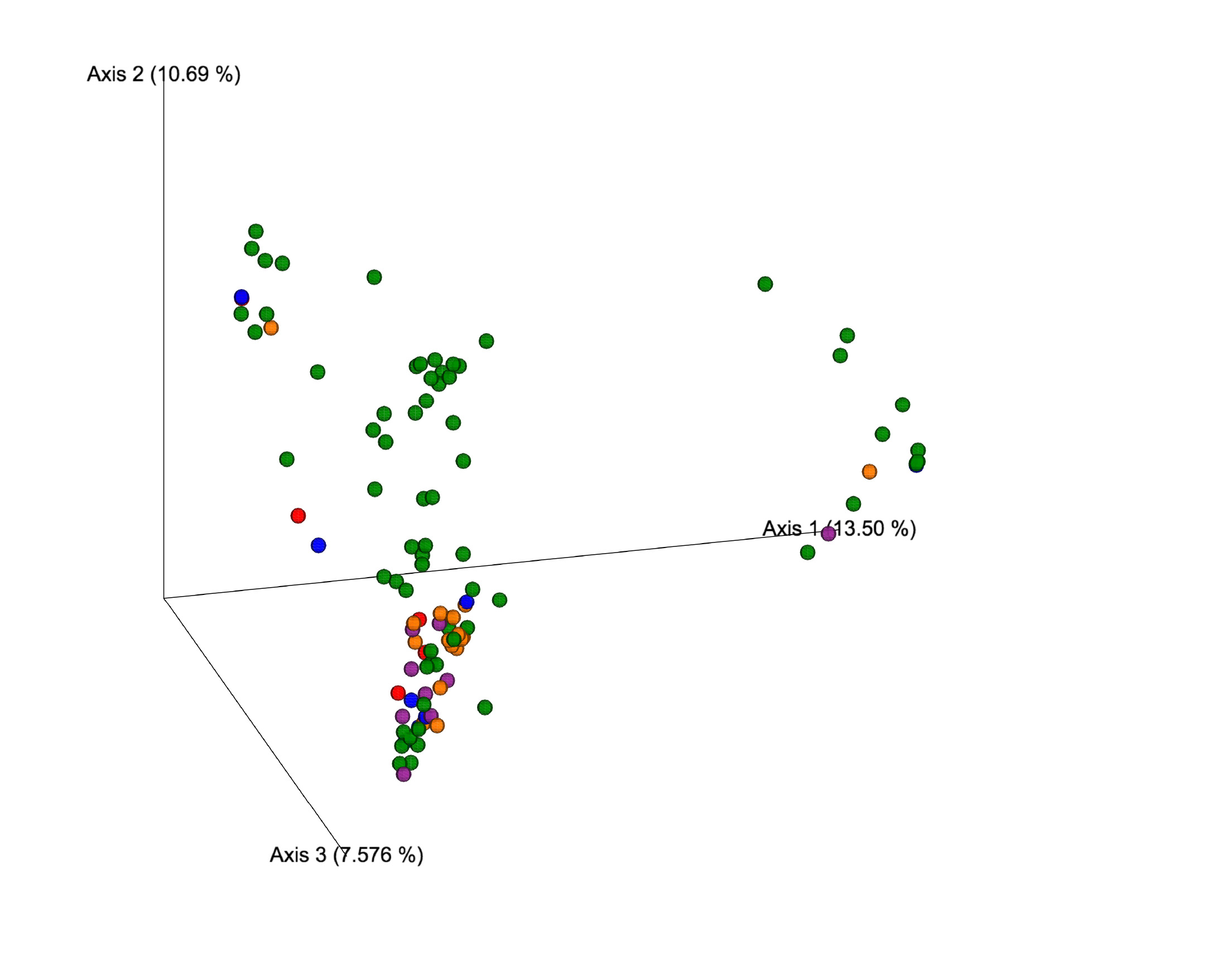

Supplement: Supplementary Figure 3 — Beta diversity plot based on abundance of OTUs in all samples of 5 groups. Green, purple, orange, blue and red represents OA, UA, IS, I and CK, respectively. [file Image_3.jpeg]

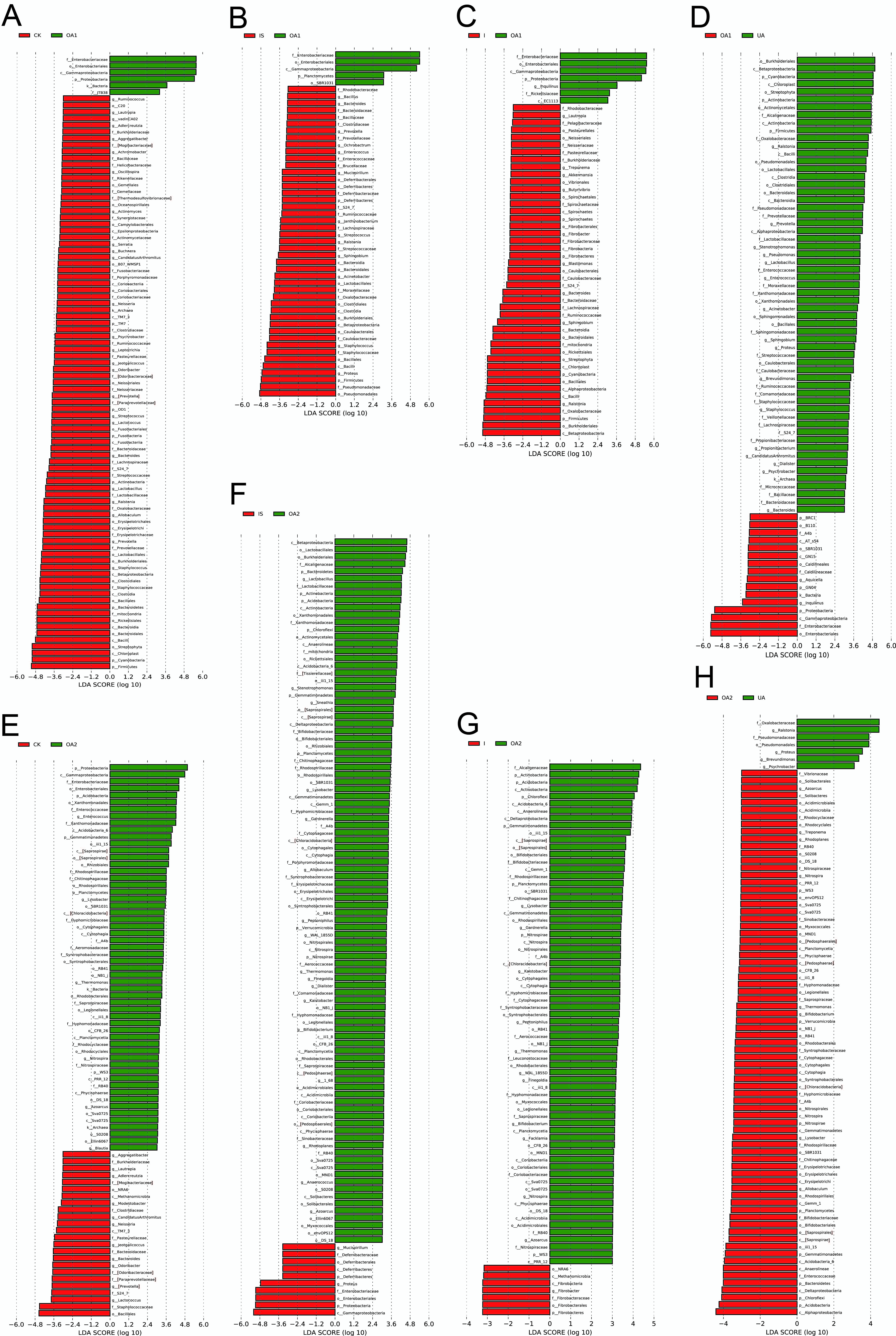

Supplement: Supplementary Figure 5 — Differentially abundant taxa for comparison of 4 groups (CA, I, IS, OA and UA) and OA1/OA2 in pairs. (A) CK and OA1; (B) IS and OA1; (C) I and OA1; (D) UA and OA1; (E) CK and OA2; (F) IS and OA2; (G) I and OA2; (H) UA and OA2. [file Image_5.jpg]
